# The relationship between gut microbiota and neonatal pathologic jaundice: A pilot case-control study

**DOI:** 10.3389/fmicb.2023.1122172

**Published:** 2023-03-16

**Authors:** Jia Jia You, Jun Qiu, Gui Nan Li, Xiao Ming Peng, Ye Ma, Chang Ci Zhou, Si Wei Fang, Rui Wen Huang, Zheng Hui Xiao

**Affiliations:** ^1^Pediatrics Research Institute of Hunan Province, Hunan Children’s Hospital, Changsha, China; ^2^Academy of Pediatrics, Hengyang Medical School, University of South China, Hengyang, China; ^3^Department of Emergency Center, Hunan Children’s Hospital, Changsha, China; ^4^Department of Neonatology, Hunan Children’s Hospital, Changsha, China

**Keywords:** *Bacteroidetes*, gut microbiota, pathologic jaundice, physiologic jaundice, neonate

## Abstract

**Background and objective:**

Neonatal jaundice is a common clinical disease in neonates. Pathologic jaundice is more harmful to neonates. There are a few studies on the biomarkers of pathologic jaundice and the correlation between gut microbiota and clinical indices. Therefore, we aimed to reveal the characteristics of gut microbiota in pathologic jaundice, provide potential biomarkers for the diagnosis of pathologic jaundice, and find the correlation between gut microbiota and clinical indices.

**Methods:**

Fourteen neonates with physiologic jaundice were recruited into a control group (Group A). Additionally, 14 neonates with pathologic jaundice were recruited into a case group (Group B). The microbial communities were analyzed using 16S rDNA sequencing. LEfSe and the differences in the relative abundance of gut microbiota were used to identify different bacteria among the two groups. The ROC curve was used to assess effective biomarkers for pathologic jaundice. Spearman’s rank-sum correlation coefficient was used to evaluate the correlation between gut microbiota and clinical indices.

**Results:**

There were no differences in the total richness or diversity of gut microbiota between the two groups. At the phylum and genus levels, compared with the control group, *Bacteroidetes* (*p* = 0.002) and *Braydrhizobium* (*p* = 0.01) were significantly higher, while *Actinobacteria* (*p* = 0.003) and *Bidfldobacterium* (*p* = 0.016) were significantly lower in the case group. *Bacteroidetes* were valuable in differentiating pathologic jaundice from physiologic jaundice by the ROC curve, and the area under the ROC curve (AUC) value was 0.839 [95%CI (0.648–0.995)]. In the case group, *Bacteroidetes* were negatively associated with total bilirubin (TBIL) (*p* < 0.05). In the control group, *Bacteroidetes* were positively associated with TBIL (*p* < 0.05).

**Conclusion:**

*Bacteroidetes* could be used as biomarkers to identify pathologic jaundice and *Bacteroidetes* are positively associated with bilirubin levels.

## 1. Introduction

Neonatal jaundice describes a condition in which an infant’s skin appears yellow by the accumulation of bilirubin (Dennery et al., 2001). It is a common clinical disease, that occurs in 80% of newborn babies (Rennie et al., 2010; Ng and How, 2015). Due to excessive generation of bilirubin, poor ability to deal with bilirubin, the insufficient serum albumin binding bilirubin ability, and increase of enterohepatic circulation influence, neonates cannot keep clear of bilirubin in the body in time, resulting in jaundice (Brits et al., 2018). Most the neonates with jaundice are relatively light conditions of physiologic jaundice (Fan and Xu, 2016). Pathologic jaundice is considered when jaundice appears early, increases too high or too fast, sustains for long terms, worsens progressively, or combines with pathologic factors (Boskabadi et al., 2020). Pathologic jaundice, if not treated in time, could cause bilirubin encephalopathy (kern jaundice) which leads to permanent damage to the nervous system and seriously affect the intellectual development and healthy growth of children (Fan and Xu, 2016). Moreover, pathologic jaundice will increase the risk of some diseases, such as childhood allergic diseases (Kuniyoshi et al., 2021), and autism spectrum disorder (Kujabi et al., 2021). Pathologic jaundice in neonates is caused by various factors, such as infection, genetic defects, and environmental factors (Akagawa et al., 2021).

Some studies have shown that the imbalance of gut microbiota, such as *Firmicutes*, *Proteobacteria*, *Bifidobacteriales*, *Escherichia*, and so on (Li et al., 2020; Akagawa et al., 2021; Ding et al., 2021), is one of the pathogenic factors of jaundice. Gut microbiota plays an important role in regulating bilirubin metabolism through enterohepatic circulation (Vitek et al., 2005). In the process of bilirubin metabolism, part of the conjugated bilirubin discharged into the intestine can be reduced to stercobilinogen and then discharged with the fecal under the action of gut microbiota. The other part can be hydrolyzed by intestinal β-glucuronidase (β-GD) to unconjugated bilirubin (Billing, 1978). Subsequently, unconjugated bilirubin can be reabsorbed through the intestinal wall by the portal vein and discharged into the intestine with bile acids after liver metabolism which forms enterohepatic circulation. However, the lack of gut microbiota transforming conjugated bilirubin in the intestine can cause the increase of bilirubin enterohepatic circulation (Sun et al., 2013), thus aggravating jaundice.

There are a few studies on the association and mechanism between gut microbiota and pathologic jaundice (Li et al., 2020). And there are a few studies on the biomarkers of pathologic jaundice and the correlation between gut microbiota and clinical indices (Zhou et al., 2019). Therefore, this study adopted the method of case–control study and the high-throughput 16S rDNA gene sequencing to detect the composition of gut microbiota in feces of neonates with physiologic jaundice and pathologic jaundice, and further reveal the characteristics of gut microbiota in neonatal pathologic jaundice, provide potential biomarkers for the diagnosis of pathologic jaundice, and find the correlation between gut microbiota and clinical indices.

## 2. Materials and methods

### 2.1. Study subjects

**Figure 1:**
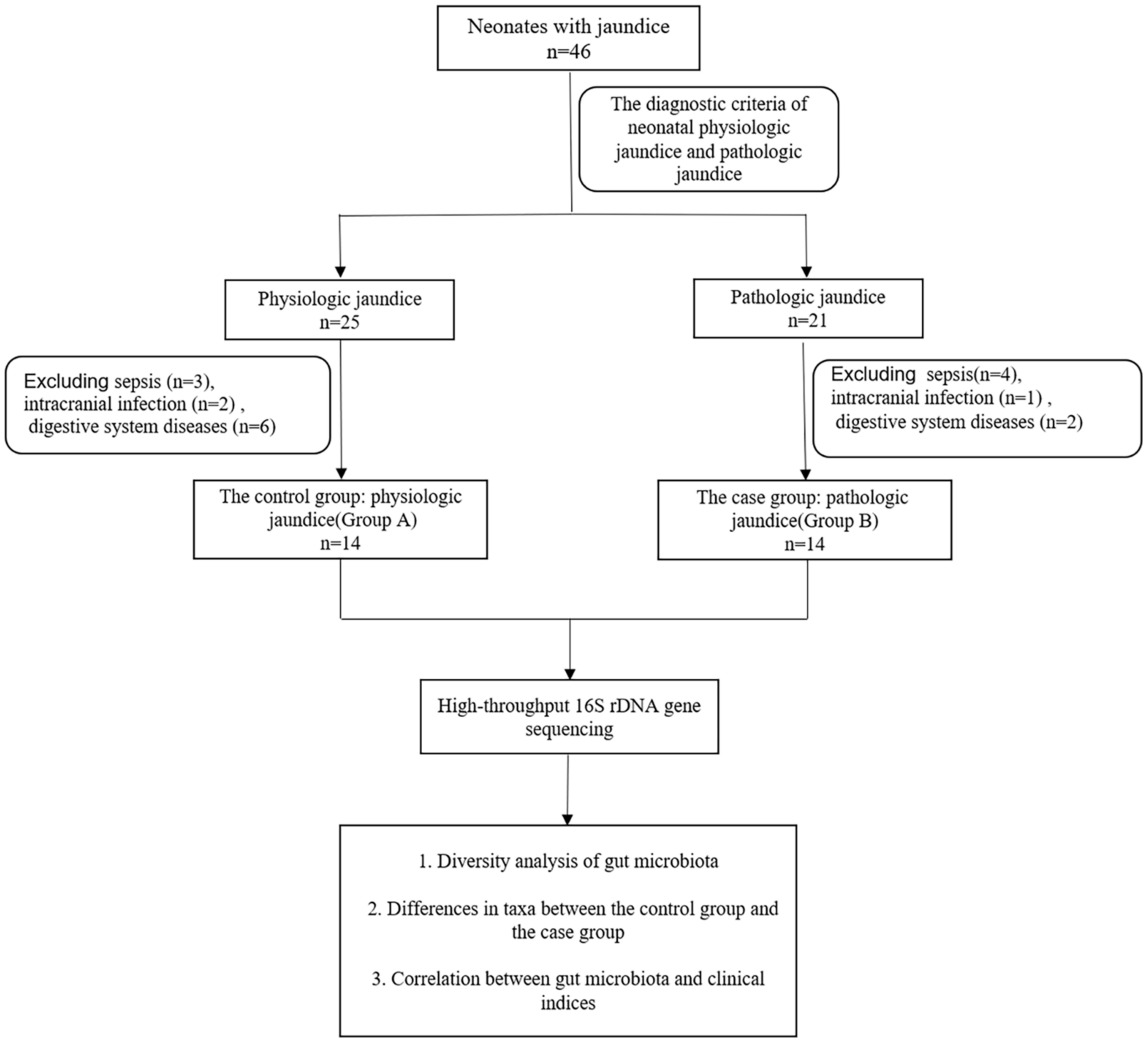
The recruitment process of all the subjects.

We selected 46 neonates with jaundice hospitalized in the Neonatology Department of Hunan Children’s Hospital from August 2018 to January 2019. According to the diagnostic criteria of neonatal physiologic jaundice and pathologic jaundice (Li and Feng, 2006; Shao et al., 2010), 25 neonates with physiologic jaundice were recruited into the control group and 21 neonates with pathologic jaundice were recruited into the case group. In the control group, three neonates with sepsis, two neonates with intracranial infection, and six neonates with digestive system diseases were excluded. Additionally, in the case group, four neonates with sepsis, one neonate with intracranial infection and, two neonates with digestive system diseases were excluded. Finally, there were 14 neonates with physiologic jaundice in the control group (Group A) and 14 neonates with pathologic jaundice in the case group (Group B). The recruitment process of all the subjects was shown in Figure 1. The study was approved by the Ethics Committee of Hunan Children’s Hospital (No. HCHLL-2018-64). Written informed consent was obtained from the parents and/or legal guardians of the enrolled children.

### 2.2. Fecal samples and general information collection

Fecal samples of neonates’ first defecation after hospitalization were collected by stool collection kit provided by Genesky Biotechnologies Inc., Shanghai, 201315 (China) and immediately frozen in ice boxes. The samples were then transported to the laboratory within 30 min and stored at −80°C. The hospitalization data of neonates were extracted from the medical record system. Collect neonates’ data of birth weightbirth weigh, age, gender, gestational age, delivery mode, the use of antibiotics, feeding status, maternal age during pregnancy, clinical diagnosis, C-reactive protein (CRP), total bilirubin (TBIL), direct bilirubin (DBIL), alanine aminotransferase (ALT), and aspartate aminotransferase (AST).

### 2.3. DNA extraction and high-throughput 16S rDNA gene sequencing

16S rDNA amplicon sequencing was performed by Genesky Biotechnologies Inc., Shanghai, 201315 (China). Total genomic DNA was extracted using the QIAamp Fast DNA Stool Mini Kit (QIAGEN ART.NO.56104). The integrity of genomic DNA was detected through agarose gel electrophoresis, and the concentration and purity of genomic DNA were detected through the Nanodrop 2000 and Qubit 3.0 Spectrophotometer. The V4–V5 hypervariable regions of the 16S rDNA gene were amplified with the primers 515F (5′-GTGCCAGCMGCCGCGG-3′) and 907R (5′-CCGTCAATTCMTTTR AGTTT-3′) (Xiong et al., 2012) and then sequenced using Illumina NovaSeq 6000 platform (Zhou et al., 2022). The sequencing data were deposited to NCBI’s Sequencing Read Archive with the accession ID PRJNA926124.

### 2.4. Gut microbial analysis

The raw read sequences were further filtered to remove adapter sequences, the primers, and low-quality reads by QIIME2 (Bolyen et al., 2019) and the cutadapt plugin to improve the accuracy of later analysis. The filtered sequences were clustered into operational taxonomic units (OTUs) of ≥97% similarity, and the sequence with the highest abundance was considered a representative sequence within each cluster (Qiu et al., 2022).

The species accumulation curve of the sample analyzed with QIIME2 was used to assess the rationality of sample content. Alpha diversity was evaluated using abundance indices and diversity indices. Chao 1 and ACE represent abundance and Shannon and Simpson represent diversity. Venn diagram which visualized with QIIME2 based on OTUs abundance was used to show the richness and similarity of gut microbiota composition within the groups. Principal component analysis (PCA) in Beta diversity (based on Bray–Curtis distance calculated with QIIME2) was used to evaluate community composition and structure of gut microbiota. Lefse analysis (Segata et al., 2011) was used to obtain species with significant differences in abundance between the control group and the case group by the linear discriminant analysis (LDA) histogram and the cladogram. The differences in the relative abundance of gut microbiota at phylum and genus levels were used to evaluate the differences in gut microbiota composition between the control group and the case group. The ROC curve calculated and displayed with R software (Version 4.1.3) was used to assess effective biomarkers for pathologic jaundice. Principal component analysis (Ringnér, 2008) based on the phylum and genus levels of gut microbiota was used to evaluate the value of their contribution to jaundice. Heatmap (Metsalu and Vilo, 2015) for Spearman’s rank-sum correlation coefficient was used to evaluate the correlation between gut microbiota and clinical indices.

### 2.5. Statistical analysis

The experimental data were analyzed by SPSS (Version 25) and R software (Version 4.1.3). For measurement data, if they were normal distribution (e.g., maternal age, Gestational age, TBIL, and Birth weight), they were expressed as mean ± SD (*X* ± *S*), and independent sample *t*-test was used for comparison difference between the two groups; if they were non-normal distribution (e.g., Age, CRP, ALT, AST, DBIL, Alpha diversity analysis index, and the relative abundance of bacteria), they were expressed as the median and interquartile range [*M* (*P*_25_,*P*_75_)], and rank sum test was used for comparison difference between the two groups. Categorical data were expressed as percentages, and the comparison differences between the two groups were evaluated by the chi-square test. The correlation between gut microbiota and clinical indices was evaluated by Spearman’s rank-sum correlation coefficient. Value of *p* < 0.05 was considered to be statistically significant.

## 3. Results

### 3.1. Clinical characteristics of the neonates

The clinical characteristics of the neonates are showed in Table 1. There were no significant differences in gender, feeding status, delivery mode, the use of antibiotics, age, maternal age, gestational age, birth weight, CRP, ALT, and AST between the two groups. The peak value of serum total bilirubin in the control group was 152.44 ± 38.24 μmol/L, and in the case group was 300.66 ± 105.97 μmol/L. There were significant differences between the two groups (*p* < 0.001).

**Table 1:** Analysis of clinical data.

	Variates	Group A (*n* = 14)	Group B (*n* = 14)	*p* value
Gender	Male	7	9	0.704
	Female	7	5	
Breastfeeding	Yes	5	6	1.000
	No	9	8	
Delivery	Eutocia	9	10	1.000
	Cesarean section	5	4	
The use of antibiotics	Yes	12	10	0.648
	No	2	4	
Age (day)		12.00 (5.00, 18.00)	10.50 (5.00, 14.25)	0.489
maternal age (year)		30.79 ± 4.74	31.21 ± 6.03	0.836
Gestational age (week)		35.51 ± 3.02	36.95 ± 2.45	0.114
Birth weight (g)		3229.29 ± 423.09	3344.29 ± 457.88	0.496
CRP (mg/L)		0.40 (0.40, 0.91)	0.40 (0.40, 0.44)	0.372
ALT (IU/L)		13.40 (10.10, 25.10)	12.35 (8.80, 15.58)	0.408
AST (IU/L)		29.85 (20.25, 34.65)	25.05 (21.22, 30.00)	0.383
TBIL (μmol/L)		152.44 ± 38.24	300.66 ± 105.97	0.000
DBIL (μmol/L)		11.80 (10.28, 14.60)	21.25 (14.88, 23.10)	0.002

CRP, C-reactive protein; ALT, alanine aminotransferase; AST, aspartate aminotransferase; TBIL, total bilirubin; and DBIL, direct bilirubin.

### 3.2. Diversity analysis of gut microbiota

**Figure 2:**
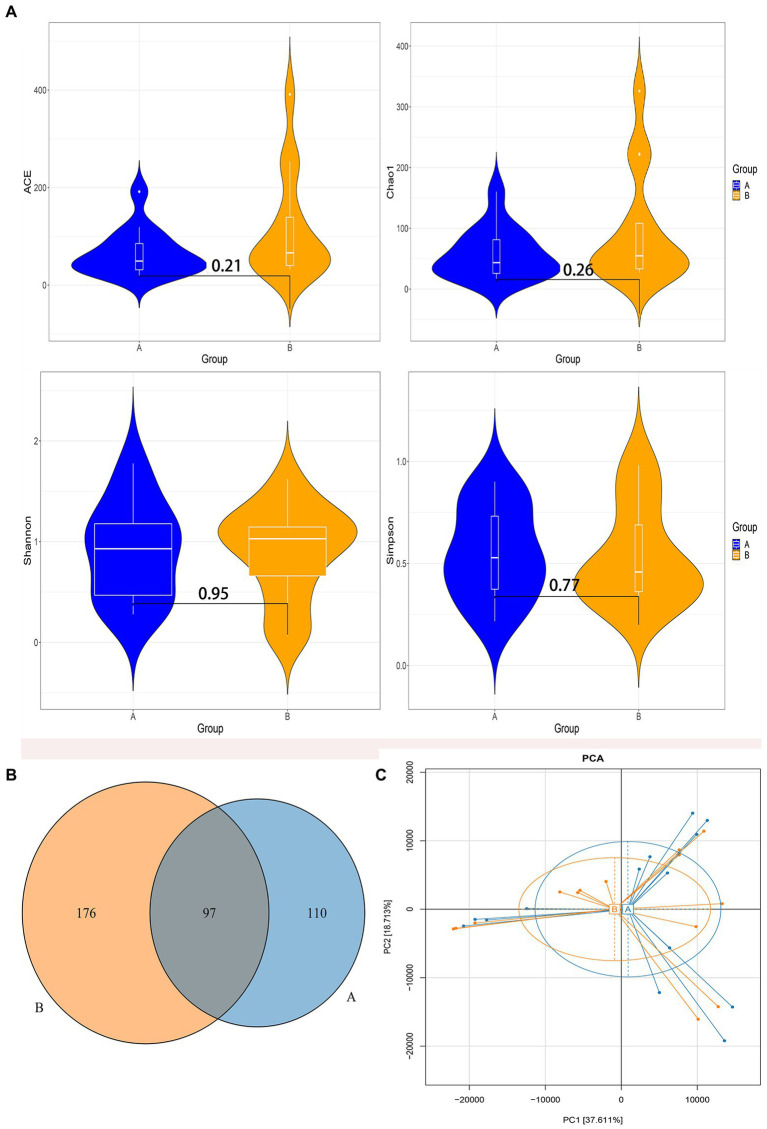
Diversity analysis of gut microbiota. **(A)** The comparison of the Alpha diversity index between the two groups. **(B)** Venn diagram of operational taxonomic units (OTUs) among the two groups. **(C)** Principal component analysis based on OTUs abundance table. Group A: the control group; Group B: the case group.

According to the species accumulation curve of the sample (Supplementary Figure 1), a total of 28 samples participated in the analysis in this study, and the sample size was sufficient, which could fully reflect the richness of the microbiota. Alpha diversity analysis among the two groups found that there were no significant differences in the alpha diversity index (Figure 2A). In the control group, the median and interquartile range of ACE index, Chao index, Shannon index, and Simpson index were 49.37 (27.93, 92.91), 43.50 (25.19, 86.55), 0.93 (0.43, 1.22), and 0.53 (0.35, 0.76), respectively. In the case group, the median and interquartile range of ACE index, Chao index, Shannon index, and Simpson index were 66.11 (38.71,169.56), 54.67 (30.43, 137.88), 1.02 (0.56, 1.18), and 0.46 (0.35, 0.76), respectively. Venn diagram showed that 97 out of 383 total OTUs were shared among the two groups (Figure 2B). Principal component analysis in Beta diversity found that the gut microbiota composition in the two groups had a high degree of similarity, and it could not be significantly distinguished from the serum bilirubin levels (Figure 2C).

### 3.3. Differences in taxa between the control group and the case group

**Figure 3:**
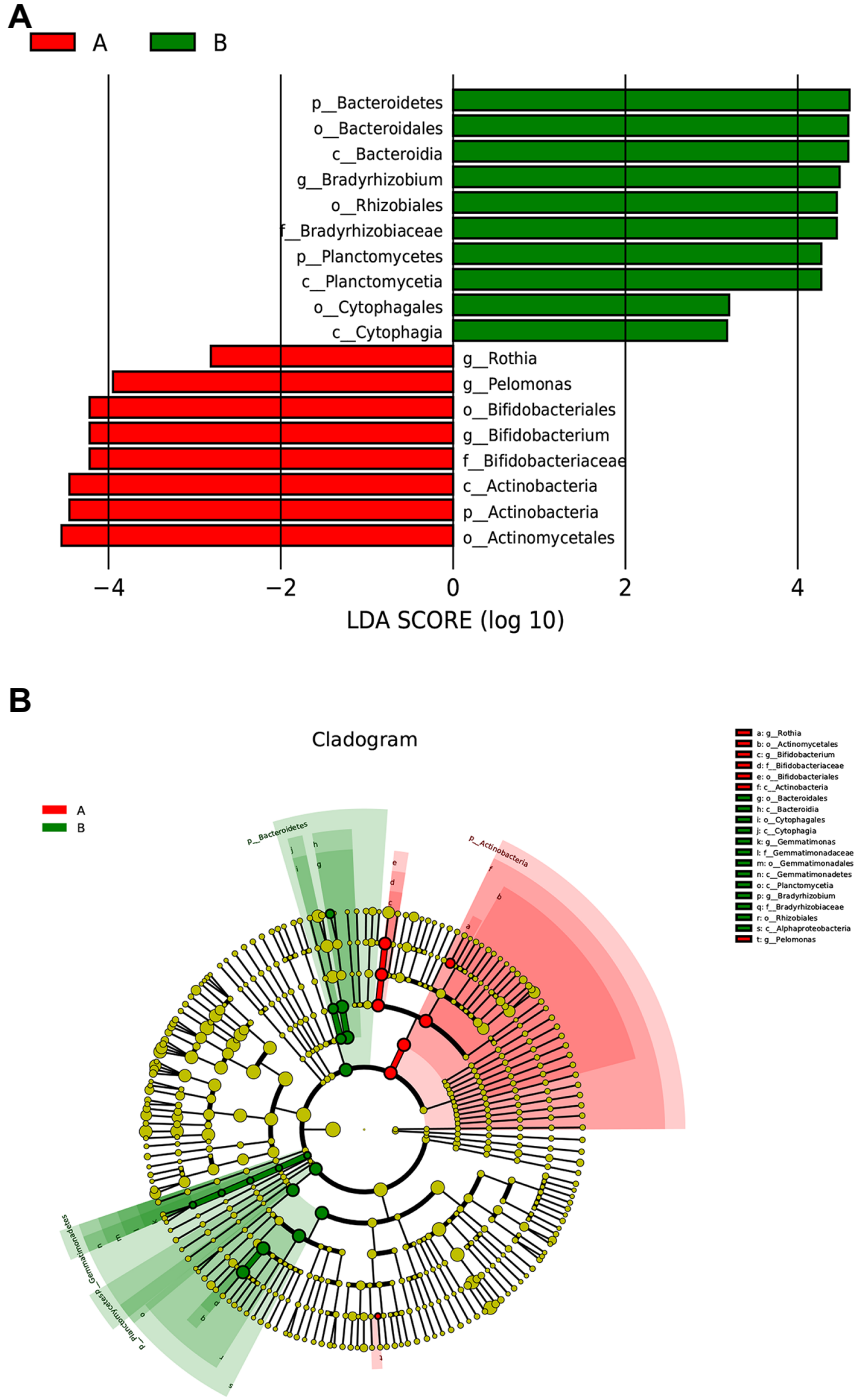
Lefse analysis between the two groups. **(A)** Histogram of LDA scores, showing significant differences in microbe type and abundance. LDA scores on the log 10 scale are indicated at the bottom. **(B)** Cladogram generated by LEfSe.

The LDA histogram showed eight bacteria (p, c, o, f, and g, respectively, representing phylum, class, order, family, and genus level) that were enriched in the fecal samples of the control group and ten bacteria that were enriched in the fecal samples of the case group (Figure 3A). *Bacteroidetes*, *Bacterodales*, *Bacteroidia*, *Braydrhizobium*, *Rhizobiales*, *Braydrhizobiaceae*, *Planctomycetes*, *Planctomycetia*, *Cytophagales*, and *Cytophagia* were abundant in the case group. As well as, *Rothia*, *pelomonas*, *Bidfldobacteriales*, *Bidfldobacterium*, *Bidfldobacteriaceae*, *Actinobacteria* (*c*), *Actinobacteria* (*p*), and *Actinomycetales* were abundant in the control group. From the LEfSe cladogram, we also found that these important bacteria were significantly abundant in the different groups (Figure 3B).

**Figure 4:**
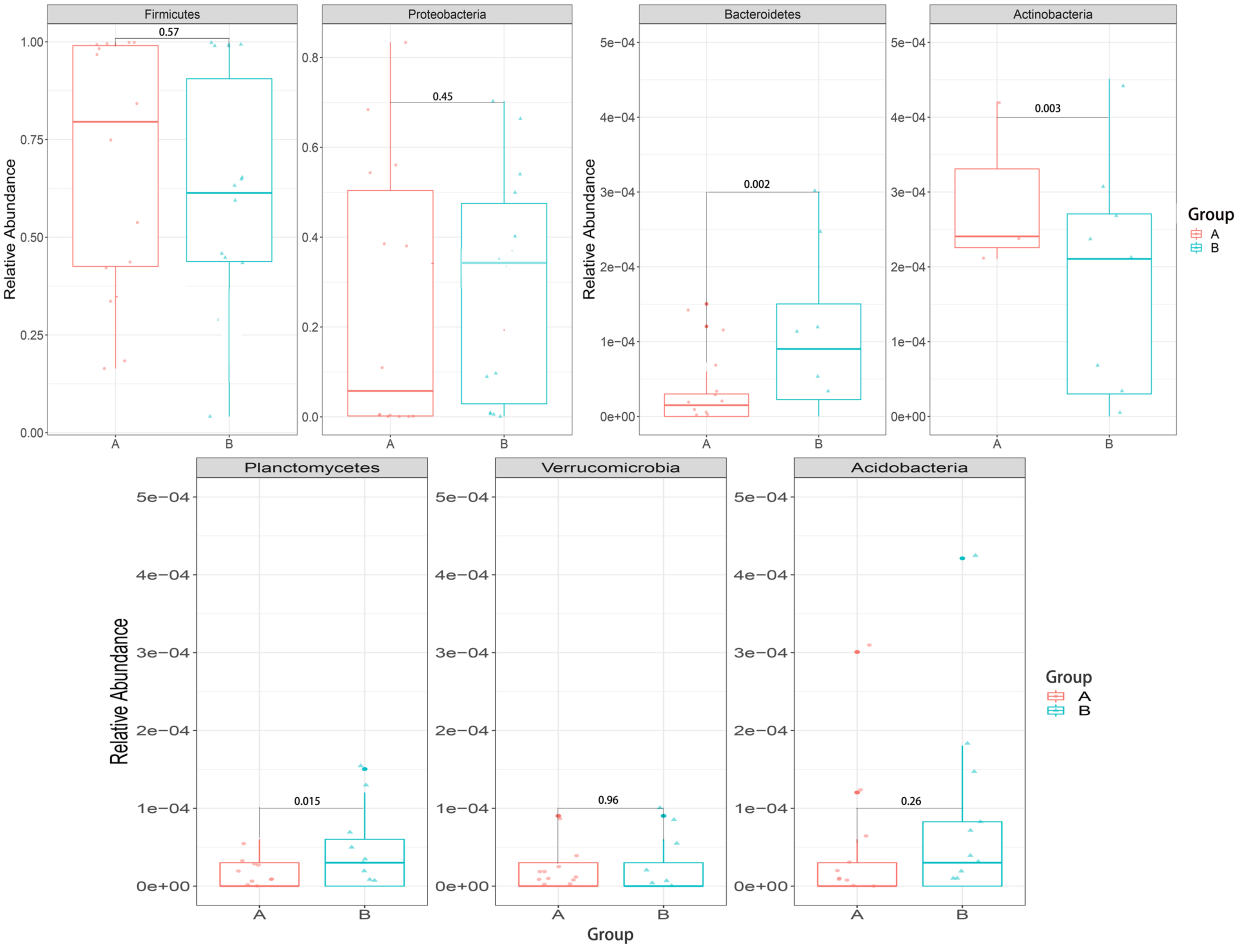
Differences in relative abundance between the two groups at the phylum level.

**Figure 5:**
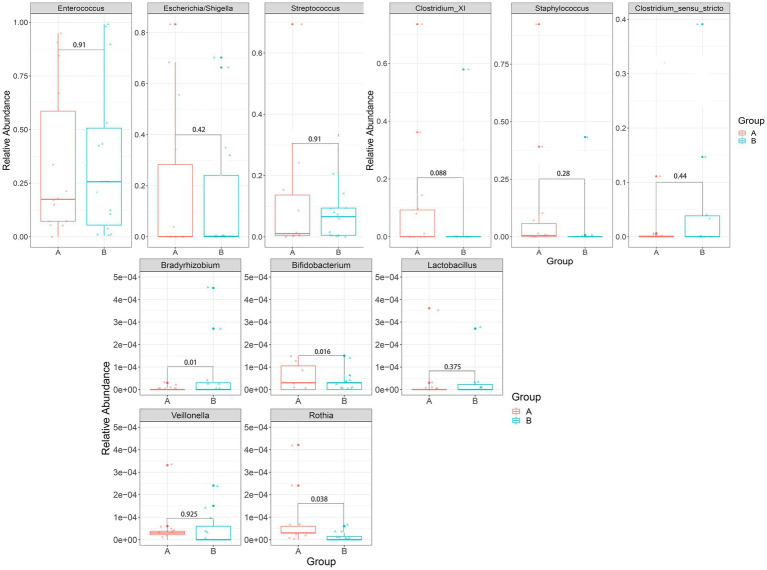
Differences in relative abundance between the two groups at the genus level.

We mainly compared the differences in the taxa at the phylum and genus levels. At the phylum level (Figure 4), compared with the control group, the relative abundance of *Bacteroidetes* [0.00027 (0.00005, 0.00830) vs. 0.00015 (0, 0.00004), *p* = 0.002] and *Planctomycetes* [0.00005 (0, 0.001889) vs. 0(0, 0.00003), *p* = 0.015] were significantly higher in the case group, while *Actinobacteria* [0.00029(0.00005, 0.00074) vs. 0.01190(0.00049, 0.09004), *p* = 0.003] were significantly lower in the case group; At the genus level (Figure 5), compared with the control group, the relative abundance of *Braydrhizobium* [0.00003(0, 0.02207) vs. 0(0,0), *p* = 0.01] was significantly higher in the case group, while *Bidfldobacterium* [0.00003 (0, 0.00006) vs. 0.0006 (0.00002, 0.04192), *p* = 0.016] and *Rothia* [0(0, 0.00017) vs. 0.00015 (0.00003, 0.00109), *p* = 0.038] were significantly lower in the case group.

**Figure 6:**
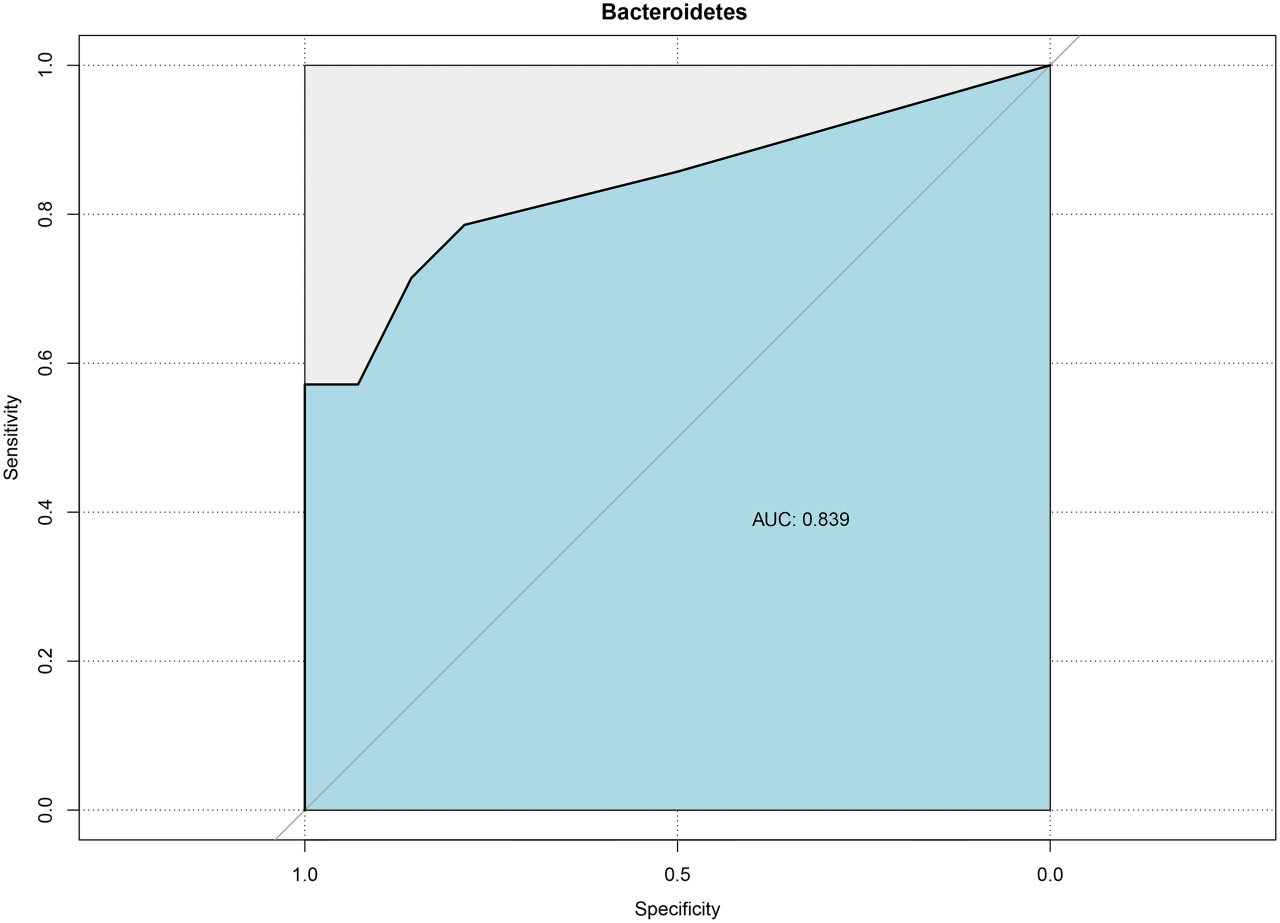
ROC curve of *Bacteroidetes* for distinguishing pathologic jaundice.

The ROC curve was used to assess effective biomarkers for pathologic jaundice (Figure 6). The results showed that a clear distinction between physiologic jaundice and pathologic jaundice could be achieved based on *Bacteroidetes* with the biggest AUC value of 0.839 [95%CI (0.648–0.995)], meaning that *Bacteroidetes* are effective biomarkers of pathologic jaundice. Besides, *Actinobacteria*, *Bidfldobacterium*, *Rothia*, *Planctomycetes*, and *Bradyrhizobium* with the area under the ROC curve (AUC) values of 0.827, 0.763, 0.724, 0.755, and 0.763 (Supplementary Figure 2).

**Figure 7:**
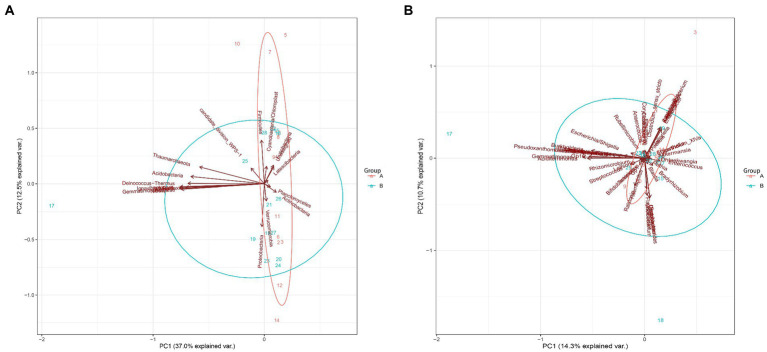
Principal component analysis between the control group and the case group at the phylum and genus levels. **(A)** The phylum level. **(B)** The genus levels.

Principal component analysis based on the phylum and genus levels of gut microbiota was used to evaluate the value of their contribution to jaundice (Figure 7). It showed that gut microbiota at phylum and genus levels could not separate the two groups. However, the differences in principal components at the phylum level were mainly contributed by *Firmicutes* and *Proteobacteria* (Figure 7A); the differences in principal components at the genus level was mainly contributed by *Gemmatimonas* (Figure 7B).

### 3.4. Correlation between gut microbiota and clinical indices

**Figure 8:**
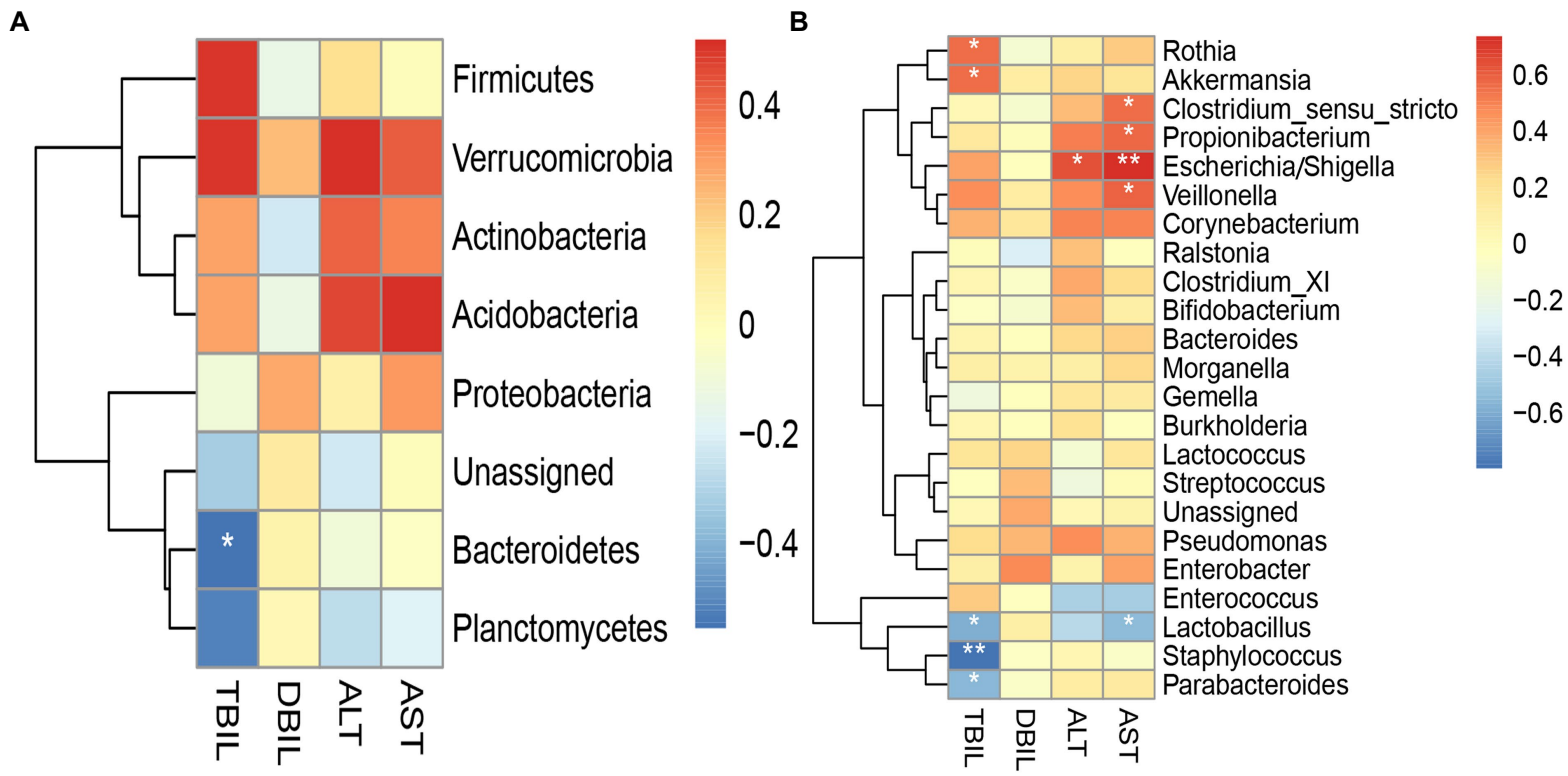
Heatmap for Spearman’s rank-sum correlation coefficient between gut microbiota and clinical indices in the case group. The red color on the heatmap indicates a positive correlation, while blue indicates a negative correlation. The darker the color, the larger the correlation coefficient. ^*^value of *p* ≤ 0.05, ^**^value of *p* ≤ 0.01. **(A)** Correlation between gut microbiota at the phylum level and clinical indices. **(B)** Correlation between gut microbiota at the genus level and clinical indices.

In the case group (Figure 8), *Bacteroidetes* and *Staphylococcus* were negatively associated with TBIL; *Escherichia/Shigella* and *Veillonella* were positively associated with AST. There were significant differences in these phenomena (*p* < 0.05). In the control group (Supplementary Figure 3), *Bacteroidetes* were positively associated with TBIL; *Pseudomonas* were positively associated with DBIL; *Acidobacteria* were negatively associated with AST; and *Rothia* was negatively associated with ALT.

## 4. Discussion

In our study, we compared the difference in gut microbiota between 14 neonates with physiologic jaundice and 14 neonates with pathologic jaundice, and then explored the correlation between gut microbiota and clinical indices. Alpha diversity analysis and Beta diversity analysis showed that there were no differences in the total richness and diversity of gut microbiota between the two groups. At the phylum and genus levels, we found that *Bacteroidetes*, *Planctomycetes*, and *Braydrhizobium* were significantly abundant in the case group, while *Actinobacteria*, *Rothia*, and *Bidfldobacterium* were significantly abundant in the control group. Then we found that *Bacteroidetes* were most valuable in differentiating pathologic jaundice from physiologic jaundice by the ROC curve. Principal component analysis based on the phylum and genus levels found that the differences in principal components at the phylum level were mainly contributed by *Firmicutes* and *Proteobacteria*. According to the heatmap for Spearman’s rank-sum correlation coefficient of the case group, *Escherichia/Shigella* and *Veillonella* were positively associated with AST. *Bacteroidetes* and *Staphylococcus* were negatively associated with bilirubin levels. And in the control group, *Bacteroidetes* were positively associated with bilirubin levels.

Our results revealed some agreement with the majority of the data that are currently published. In our study, Alpha diversity analysis showed that there were no differences in the total richness and diversity of gut microbiota between the two groups. Akagawa et al. found that there were no significant differences in the total richness and diversity of gut microbiota between 26 neonates with jaundice and 17 neonates without jaundice through 16S rRNA gene sequencing (Akagawa et al., 2021). Beta diversity analysis showed that there was a greater similarity in gut microbiota between the two groups. Dong et al. using 16S rRNA gene sequencing found there were no differences in Beta diversity of gut microbiota between 141 children with jaundice and 160 children without jaundice (Dong et al., 2018). This may suggest that the diversity of gut microbiota is not associated with jaundice severity.

Lefse analysis found that *Bacteroidetes*, *Planctomycetes*, and *Braydrhizobium* were abundant in the case group, while *Actinobacteria*, *Rothia*, and *Bidfldobacterium* were abundant in the control group. Meanwhile, at the phylum and genus levels, we found that the *Bacteroidetes*, *Planctomycetes*, and *Braydrhizobium* were significantly higher and *Actinobacteria*, *Rothia*, *and Bidfldobacterium* were significantly lower in the case group. Zhou et al. found that *Bacteroidetes* significantly increased in neonates with cholestasis jaundice by shotgun metagenomic sequencing (Zhou et al., 2019), which was consistent with our study. Besides, there was a study finding that *Bacteroides* were higher in patients with cholestatic liver disease (Wu et al., 2020). *Bacteroides* have both positive and negative effects on the human body (Zafar and Saier, 2021). On the one hand, *Bacteroides* can ferment carbohydrates in the gut, producing fatty acids that provide an energy source for the host (Hooper et al., 2002). Moreover, *Bacteroides thetaiotaomicron* of *Bacteroidetes* can regulate the expression of some host genes, including those associated with nutrient absorption, mucosal barrier fortification, and production of angiogenic factors (Hooper et al., 2001). Among them, it plays an important role in maintaining the structure and function of the intestinal mucosal barrier (Martin-Gallausiaux et al., 2021). *Bacteroides thetaiotaomicron* is reported to induce the expression of the small proline-rich protein 2A (sprr2A), which is necessary to maintain the epithelial villus desmosomes (Lutgendorff et al., 2008). Additionally, *B. thetaiotaomicron* can produce an antibiotic Paneth cell protein (Ang4) that can kill certain pathogenic organisms (Wexler, 2007). One the other hand, *Bacteroides fragilis* of *Bacteroidetes* has the ultra-high ability to utilize nearby nutrients. It can use glycoproteins and glycolipids on the surface of host cells at the site of infection to compete for the utilization of human nutrients. Besides, *B. fragilis* has harmful effects on the human body by participating in tissue adhesion, preventing host immune response, and destroying tissue components (Wexler, 2007). Previous studies have shown that *B. fragilis* accounted for 50% of the pathogenic bacteria in neonatal bacteremia (Brook, 1990). Also, some studies have noted that the increased abundance of *Bacteroides* can cause chronic inflammatory effects and lead to the development of type 1 diabetes mellitus (T1DM), celiac disease and other diseases (Torun et al., 2021). In our study, *Bacteroides* were more abundant in the case group, which may cause intestinal inflammation most likely due to the increase of harmful bacteria *B. fragilis*. However, we could not identify the certain bacteria species due to technical limitation.

*Braydrhizobium* belongs to *Proteobacteria*, and *Proteobacteria* belongs to anaerobes or facultative anaerobes. The colonic epithelium is hypoxic, which indicates that there were changes in intestinal epithelium and mucosa in neonates with pathologic jaundice (Li et al., 2020). Studies have noted that there was severe impairment of intestinal mucosal barrier function in patients with obstructive jaundice (Parks et al., 1996). Akagawa et al. found that *Bidfldobacterium* significantly decreased in neonates with jaundice (Akagawa et al., 2021). Zhou et al. also found that *Bidfldobacterium* significantly decreased in neonates with cholestasis jaundice by shotgun metagenomic sequencing (Zhou et al., 2019). *Bidfldobacterium belongs* to *Actinobacteria.* In the process of galactooligosaccharides (GOS) utilization, *Bifidobacteriales* can convert GOS into galactose and UDP-glucose, and the latter can affect the glucuronic acid pathway in the liver. So, the reduction of *Bifidobacteria* can affect the production of conjugated bilirubin (Thongaram et al., 2017; Duan, 2018). *Rothia* also belongs to *Actinobacteria*. It can produce butyrate (Liang et al., 2021). Butyrate belongs to short-chain fatty acids (SCFAs) which can regulate inflammation and reduce the number of inflammatory macrophages and Th17 cells in the intestine (Jiang et al., 2016). It has been reported that SCFAs may lead to changes in the gut microbiota to some extent (Zheng et al., 2017). Duan et al. found that SCFAs significantly decreased in neonates with breast milk jaundice. Therefore, they hypothesized that the alteration of gut microbiota may affect the production of SCFAs, and then SCFAs affect bilirubin metabolism (Duan et al., 2021). Furthermore, the flagella of *Rothia* can repair the intestinal barrier, improve the intestinal ecosystem, and alleviate chronic inflammation (Seo et al., 2020). Therefore, it can be concluded that *Bacteroidetes*, *Actinobacteria*, *Braydrhizobium*, *Rothia*, and *Bifidobacterium* may affect bilirubin metabolism by causing intestinal inflammation, changing intestinal mucosal permeability, affecting glucuronic acid metabolism pathway, and antagonizing intestinal inflammation. By the ROC curve, we found that *Bacteroidetes*, *Actinobacteria*, *Bifidobacterium*, *Rothia*, *Planctomycetes*, and *Bradyrhizobium* had the significance of distinguishing pathologic jaundice from physiologic jaundice. Interestingly, *Bacteroidetes* were most valuable in differentiating pathologic jaundice from physiologic jaundice. Therefore, in the future clinical work, *Bacteroidetes* could be used as biomarkers to identify pathologic jaundice. At the same time, it could be used to determine whether the etiology of jaundice is endogenous or intestinal to a certain extent (Guo, 2017).

This study found that the differences in principal components at the phylum level were mainly contributed by *Firmicutes* and *Proteobacteria*. Studies have shown that gut microbiota in the neonatal period is mainly composed of *Firmicutes* and *Proteobacteria* (Jiménez et al., 2008). *Firmicutes* can produce butyrate (Fei et al., 2018), which plays an important role in intestinal conjunctivitis. It is the main energy source of the intestinal mucosa, and plays an important role in inhibiting colonic carcinogenesis, resisting inflammation, regulating oxidative stress, improving colonic defense barrier function, and promoting satiety (Hamer et al., 2008; Louis and Flint, 2009). By studying the changes in gut microbiota in rats with non-alcoholic fatty liver disease (NAFLD) model, Sun found that *Proteobacteria* significantly increased and were positively associated with serum aspartate aminotransferase (Sun, 2020). Previous studies have shown that *Proteobacteria* are related to the integrity of the intestinal mucosal barrier, and the increased abundance of *Proteobacteria* destroys the original intestinal mucosal barrier and increases intestinal permeability, thereby causing chronic intestinal inflammation and liver function damage (Jiang et al., 2015; Sun, 2020). In our study, *Firmicutes* decreased and *Proteobacteria* increased in the pathologic jaundice group, although there were no significant differences. The imbalance of *Proteobacteria* and *Firmicutes* may change intestinal permeability and cause chronic intestinal inflammation, then impair liver function and reduce liver processing capacity for bilirubin.

In the case group, the heatmap for Spearman’s rank-sum correlation coefficient showed that *Escherichia/Shigella* and *Veillonella* were positively associated with AST, while *Bacteroidetes* and *Staphylococcus* were negatively associated with bilirubin levels. Wu et al. found that *Escherichia/Shigella* and *Veillonella* increased in patients with cholestatic liver disease by 16S rRNA gene sequencing. *Escherichia/Shigella* and *Veillonella* are opportunistic pathogens, whose abundance will increase under the alteration of gut microbiota and eventually become the dominant bacterial genus in pathologic jaundice (Shen et al., 2017; Wu et al., 2020). The relatively high abundance of *Veillonella* might be used as a potential biomarker for the diagnosis of cholestatic liver disease (Wu et al., 2020). As we mentioned above, *B. fragilis* of *Bacteroidetes* may increase serum bilirubin levels by causing intestinal inflammation, and changing intestinal mucosal permeability. Some studies have shown that *Staphylococcus* can produce β-glucuronidase (β-GD) (Tang et al., 2021). In the intestine, β-GD can hydrolyze conjugated bilirubin into unconjugated bilirubin. Then, unconjugated bilirubin is absorbed by the intestinal mucosa, coming back to the liver, thereby promoting the serum bilirubin levels (Seyedi et al., 2019). And β-GD activity is positively associated with the abundance of *Staphylococcus* (Tang et al., 2021). However, this is contrary to the results of our experiment, which may be due to the fact that the bilirubin levels in the case group were determined by two factors. On the one hand, some neonates were recruited into the case group due to high bilirubin levels. One the other hand, the left neonates were recruited into the case group due to prolonged jaundice. So, we got a result that *Bacteroidetes* were positively associated with bilirubin levels in the control group. Because the diagnosis of physiologic jaundice is simply elevated bilirubin levels. It can be concluded that *Escherichia/Shigella* and *Veillonella* may become the dominant pathogens due to the imbalance of gut microbiota, causing changes in liver function. *Bacteroidetes* cause intestinal inflammation most likely due to the harmful bacteria *B. fragilis*, while *Staphylococcus* increases enterohepatic circulation by producing β-GD, which will cause an elevated serum bilirubin level.

There were some limitations in our study. Firstly, in this study, the control group recruited the subjects with physiologic jaundice, while the other studies mentioned above recruited the subjects with non-jaundice. There may be some differences in the gut microbiota between the physiologic jaundice group and the non-jaundice group. It is less supportive when discussing the pathogenicity with other studies. Secondly, although there were no significant differences in CRP between the two groups by baseline comparison. However, the individual subject has elevated CRP values, which may affect the development of gut microbiota. So, the confounding effects of infection have not been excluded. In the next step, healthy neonates will be selected as the research subjects. Additionally, although there was no difference in the rate of antibiotic use between the two groups in our study, antibiotic use can have an impact on gut microbiota. In the future studies about gut microbiota, we will study children who have not received antibiotics. Finally, we failed to follow up and continue to collect specimens for a longitudinal study to observe the changes and characteristics of gut microbiota.

## 5. Conclusion

*Bacteroidetes*, *Planctomycetes*, and *Braydrhizobium* were significantly abundant in the case group, while *Actinobacteria*, *Rothia*, and *Bidfldobacterium* were significantly abundant in the control group. *Bacteroidetes* were valuable in differentiating pathologic jaundice from physiologic jaundice by the ROC curve. Therefore, *Bacteroidetes* could be used as biomarkers to identify pathologic jaundice. Moreover, *Bacteroidete*s are positively associated with bilirubin levels. It affects bilirubin metabolism by causing intestinal inflammation most likely due to the harmful bacteria *B. fragilis.*

## Data availability statement

The datasets presented in this study can be found in online repositories. The names of the repository/repositories and accession number (s) can be found at: https://www.ncbi.nlm.nih.gov/sra/PRJNA926124, PRJNA926124.

## Ethics statement

The studies involving human participants were reviewed and approved by The Ethics Committee of Hunan Children’s Hospital (Changsha, China. No. HCHLL-2018-64). Written informed consent to participate in this study was provided by the participants’ legal guardian/next of kin.

## Author contributions

JY and JQ contributed to the conception and design of the work and revised the manuscript for important intellectual content. RH, ZX, and JQ provided administrative support. GL, XP, YM, RH, and ZX provisioned the study materials or patients. JY, CZ, and YM collected and assembled the data. JY, SF, CZ, and JQ performed the statistical analysis and interpretation. JY drafted the manuscript. All authors contributed to the article and approved the submitted version.

## Funding

This research was funded by the Science and Technology Department of Hunan Province (2020SK1014-3).

## Conflict of interest

The authors declare that the research was conducted in the absence of any commercial or financial relationships that could be construed as a potential conflict of interest.

## Publisher’s note

All claims expressed in this article are solely those of the authors and do not necessarily represent those of their affiliated organizations, or those of the publisher, the editors and the reviewers. Any product that may be evaluated in this article, or claim that may be made by its manufacturer, is not guaranteed or endorsed by the publisher.
