# Supplementary material for: The relationship between gut microbiota and neonatal pathologic jaundice: A pilot case-control study
Source: Front Microbiol. 2023 Mar 16;14:1122172. doi: 10.3389/fmicb.2023.1122172 (PMC10060978; doi:10.3389/fmicb.2023.1122172)
Supplement: Supplementary file 1 [file Data_Sheet_1.docx]

Supplementary Material

**The relationship between gut microbiota and neonatal pathologic jaundice: A pilot case-control study**

Jiajia You ^1, 2, 3^ ^†^, Jun Qiu ^1^ ^†^, Guinan Li ^4^, Xiaoming Peng ^4^, Ye Ma^4^, Changci Zhou ^1, 2, 3^, Siwei Fang^1, 2, 3^, Ruiwen Huang^4*^, Zhenghui Xiao ^3*^

^1^Pediatrics Research Institute of Hunan Province, Hunan Children's Hospital, Changsha, China

^2^Academy of Pediatrics, Hengyang Medical School, University of South China, Hengyang, China

^3^Department of Emergency Center, Hunan Children's Hospital, Changsha, China

^4^Department of Neonatology, Hunan Children's Hospital, Changsha, China

*** Correspondence:**Ruiwen Huang, Email: 846767295@qq.com

Zhenghui Xiao, Email: xiaozh888@126.com

^†^These authors contributed equally to this work and share first authorship

# Supplementary Figures


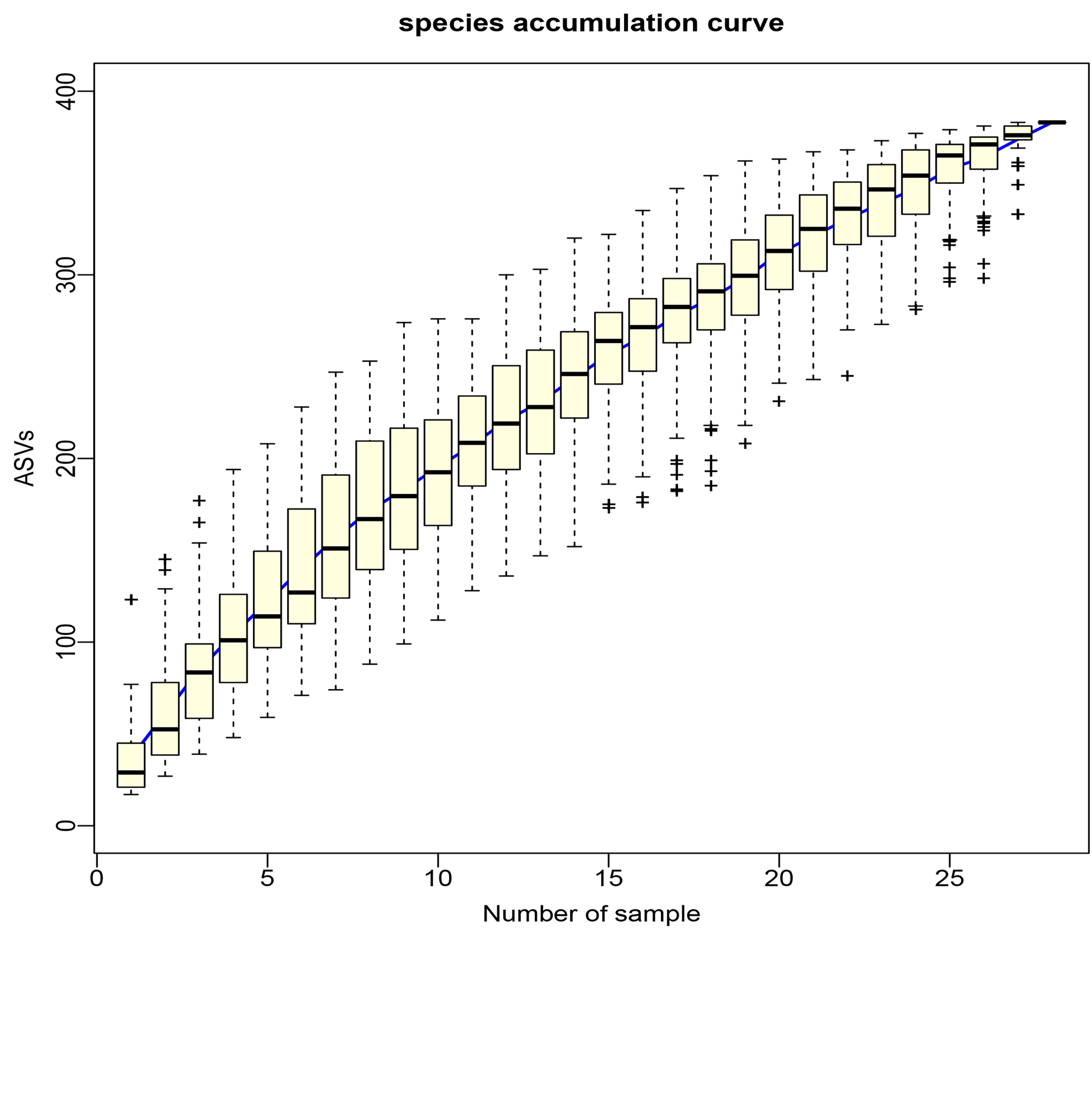


**Supplementary Figure 1.** Species accumulation curve of the sample.





**Supplementary Figure 2**. ROC curve of bacteria for distinguishing pathologic jaundice from physiologic jaundice.





**Supplementary Figure 3.** Heatmap for Spearman’s rank-sum correlation coefficient between gut microbiota and clinical indices in the control group.
